# Empirical use of antibiotics and adjustment of empirical antibiotic therapies in a university hospital: a prospective observational study

**DOI:** 10.1186/1471-2334-7-21

**Published:** 2007-03-26

**Authors:** Julian Mettler, Mathew Simcock, Pedram Sendi, Andreas F Widmer, Roland Bingisser, Manuel Battegay, Ursula Fluckiger, Stefano Bassetti

**Affiliations:** 1 Division of Infectious Diseases & Hospital Epidemiology, University Hospital Basel, CH – 4031 Basel, Switzerland; 2 Basel Institute for Clinical Epidemiology, University Hospital Basel, CH – 4031 Basel, Switzerland; 3 Emergency Department, University Hospital Basel, CH – 4031 Basel, Switzerland; 4 Department of Internal Medicine, Kantonsspital Olten, Baslerstrasse 150, CH – 4600 Olten, Switzerland

## Abstract

**Background:**

Several strategies to optimise the use of antibiotics have been developed. Most of these interventions can be classified as educational or restrictive. Restrictive measures are considered to be more effective, but the enforcement of these measures may be difficult and lead to conflicts with prescribers. Any intervention should be aimed at targets with the highest impact on antibiotic prescribing. The aim of the present study was to assess the adequacy of empirical and adjusted antibiotic therapies in a Swiss university hospital where no antibiotic use restrictions are enforced, and to identify risk factors for inadequate treatment and targets for intervention.

**Methods:**

A prospective observational study was performed during 9 months. All patients admitted through the emergency department who received an antibiotic therapy within 24 hours of admission were included. Data on demographic characteristics, diagnoses, comorbidities, systemic inflammatory response syndrome (SIRS) parameters, microbiological tests, and administered antibiotics were collected prospectively. Antibiotic therapy was considered adequate if spectrum, dose, application modus, and duration of therapy were appropriate according to local recommendations or published guidelines.

**Results:**

2943 admitted patients were evaluated. Of these, 572 (19.4%) received antibiotics within 24 hours and 539 (94%) were analysed in detail. Empirical antibiotic therapy was inadequate in 121 patients (22%). Initial therapy was adjusted in 168 patients (31%). This adjusted antibiotic therapy was inadequate in 46 patients (27%). The main reason for inadequacy was the use of antibiotics with unnecessarily broad spectrum (24% of inadequate empirical, and 52% of inadequate adjusted therapies). In 26% of patients with inadequate adjusted therapy, antibiotics used were either ineffective against isolated pathogenic bacteria or antibiotic therapy was continued despite negative results of microbiological investigations.

**Conclusion:**

The rate of inadequate antibiotic therapies was similar to the rates reported from other institutions despite the absence of a restrictive antibiotic policy. Surprisingly, adjusted antibiotic therapies were more frequently inappropriate than empirical therapies. Interventions aiming at improving antibiotic prescribing should focus on both initial empirical therapy and streamlining and adjustment of therapy once microbiological results become available.

## Background

Antibiotic resistance of bacteria is an increasing, world-wide problem [1-3]. The use of antibiotics is an important factor contributing to the emergence of antibiotic-resistant bacteria [4,5], and it is well known, that a large proportion of administered antibiotics is prescribed without proper indication [6-11]. Published rates of inappropriate antibiotic use are as high as 41% to 91% [6,11].

Several strategies to optimise the use of antibiotics, often referred to as antibiotic stewardship programs, have been developed. These interventions can be classified into two main categories: educational, and restrictive or coercive [12]. Both types of intervention may be useful in reducing the amount of prescribed antibiotics and costs of therapy while maintaining quality of care [13,14]. However, restrictive measures are considered to be more effective and to have a longer lasting impact than educational strategies [12].

The aim of the present study was to assess the adequacy of empirical and adjusted antibiotic therapies at a Swiss university hospital where no restrictive measures regarding antibiotic use are in place, and to identify risk factors for inadequate treatment and targets for intervention.

## Methods

The study was conducted prospectively during a period of 9 months (November 17, 2003 – July 31, 2004) on alternating weeks at the University Hospital Basel, a 700-bed hospital in Northwestern Switzerland providing primary and tertiary care for adult patients. The emergency department of this hospital handles all emergencies except gynecological/obstetrical, ophthalmologic and pediatric ones. In 2004, 38.5% of all in-patients were admitted through the emergency department.

Antibiotics available within the hospital are listed in the drug formulary. All drugs included in the formulary can be prescribed without restrictions. In addition, the hospital pharmacy provides drugs which are not in the formulary upon request, although physicians are encouraged to use listed agents. Written local recommendations for the antibiotic treatment of the most common infectious diseases are available to all hospital physicians on the intranet and as a booklet. These internal guidelines include recommendations on diagnosis and treatment of pneumonia, sepsis, endocarditis, urinary tract infections, infections of the central nervous system, intravascular catheter-related infections, fever in neutropenic patients, and on empirical therapy in patients with suspected infection. The guidelines focus mainly on the choice and on the dose of the antibiotic. Recommendations on duration of therapy (and on duration of i.v. therapy) are provided for some indications (e.g. endocarditis, meningitis, intravascular catheter-related infections, urinary tract infections). There are no general recommendations for the switch from i.v. to p.o. An active infectious diseases consultation service is present in the hospital and has a close collaboration with the microbiology laboratory. In addition, weekly infectious diseases rounds are held on all medicine wards, on the bone marrow transplant unit and on the intensive care units.

All patients admitted to the University Hospital Basel through the emergency department during the mentioned period were included in the study and prospectively followed-up for the use of therapeutic antibiotics. Data of all patients receiving an antibiotic therapy within 24 hours of admission were further analysed. These patients began an antibiotic treatment within 24 hours of admission or continued an antibiotic treatment started in another hospital. They were treated as in-patients on the emergency department ward, or transferred to the departments of internal medicine (including neurology and geriatrics), surgery, or to an intensive care unit, either directly after admission, or subsequently from the emergency department ward. The following patients were excluded: patients admitted to the eight-bed acute psychiatric intervention unit, to dermatology, ophthalmology, gynecology/obstetrics, or otorhinolaryngology wards; patients given antibiotics for prophylaxis or for *Helicobacter pylori *eradication; patients who started antibiotic therapies as out-patients and patients transferred to another hospital within 48 hours of admission.

Information on demographics, admission diagnoses, empirical and adjusted antibiotic therapies, duration of therapy, specimens submitted for bacteriological testing, results of microbiological investigations, involvement of infectious diseases consultants, Charlson comorbidity index [15], McCabe index [16] and systemic inflammatory response syndrome (SIRS) parameters were prospectively recorded on a standardised case report form. After discharge, all prospectively collected data were verified and completed by chart review. In addition, data on microbiological investigations were retrieved from the microbiology lab's internal computer system. Each case report form with the corresponding chart was reviewed together by two of the authors (J.M., S.B.), who assessed the adequacy of empirical and adjusted antibiotic therapy by consensus. Prescribing was reviewed daily and the appropriateness of the antibiotic, the dose and route of administration were assessed at any time during the course of therapy.

The empirical antibiotic therapy was defined as the initial antibiotic regimen started within 24 hours of admission. The adjusted antibiotic therapy was the antibiotic regimen after the first change of antibiotic substance. The switch of the same antibiotic from i.v. to p.o. was not considered to be an adjustment of therapy. Finally, we defined an antibiotic therapy as inadequate when one or more of the following points were not in accordance with local written recommendations or published guidelines (e.g. The Sanford Guide to Antimicrobial Therapy, practice guidelines of the Infectious Diseases Society of America): spectrum, dosage, application modus of antibiotics, or the duration of therapy, and/or when pathogenic bacteria that were resistant to the antibiotic used were isolated. If the application modus was considered to be not appropriate, the reason was recorded as "insufficient dosage" (e.g. if a patient with catheter-related bloodstream infection caused by *S. aureus *was treated with amoxicillin/clavulanate p.o.), or "excessive dosage" (e.g. if a patient with uncomplicated pneumonia caused by susceptible *S. pneumoniae *was kept on penicillin i.v.).

### Statistical Methods

For the univariate analyses, t-tests between continuous variables and Chi-squared tests (or Fisher's Exact tests when appropriate) between categorical variables were used. All associations found to be statistically significant at the 25% significance level in the univariate analysis were collectively considered for the multivariate analysis to produce a model for both the empirical antibiotic therapy and adjusted antibiotic therapy separately. The multivariate analysis used a logistic regression model with an underlying binomial distribution with the Logit link. The goodness of fit of the model was assessed with the Log likelihood ratio test. The covariates considered for the empirical antibiotic therapy multivariate analysis were gender, age, neurological ward, involvement of infectious disease consultants, renal failure, use of broad-spectrum antibiotics, and superficial skin infections. For the adjusted antibiotic therapy multivariate analysis the following were considered: switch of antibiotic therapy due to bacteriological lab results, involvement of infectious disease consultants and patients with respiratory tract infections; however, none of these variables were found to be of importance in the final model. Confounders and plausible interactions considered were found not to be statistically significant for entry into the model. All statistical analyses were carried out using SAS version 8.2 (SAS Institute Inc., Cary, NC, USA).

The study was approved by the Research Ethics Committee of the Cantons Basel-Stadt and Basel-Land (N. 226/03).

## Results

During the nine-month study period a total of 7792 patients were admitted to the University Hospital Basel through the emergency department. Of these patients, 3387 were admitted during the weeks when the study was carried out. Referral to another hospital within 48 hours after admission led to the exclusion of 224 patients. Referral to the psychiatry, dermatology, gynecology/obstetrics or otorhinolaryngology wards led to the exclusion of 88 patients, and 132 patients were excluded since they were given therapeutic antibiotics prior to admission in an out-patient setting (all these 132 patients had received antibiotics p.o.). A total of 572 of the remaining 2943 patients (19.4%) were given therapeutic antibiotics within 24 hours after admission. Incomplete or missing records led to the exclusion of 33 patients. The remaining 539 patients (94.2%) were analyzed. Data on demographics, infectious diseases diagnosed on admission, and the service in charge of these patients are presented in Table 1.

**Table 1:** Characteristics of the 539 included patients.

Characteristic	Number of patients (n = 539)	Percentage of all patients
Males	297	55.1%
Age in years (median; range)	69 [17–100]	
17 to 40 years	96	17.8%
41 to 60 years	104	19.3%
More than 60 years	339	62.9%
Ward ^a^		
Medicine and geriatrics	359	66.6%
Surgery	173	32.1%
Medical intensive care	25	4.6%
Surgical intensive care	10	1.9%
Neurology	4	0.7%
Main infectious disease diagnosis upon admission		
Respiratory tract infections	169	31.4%
Urinary tract infections, prostatitis, epididimytis	113	21.0%
Gastrointestinal infections ^b^	81	15.0%
Skin and soft tissue infections	42	7.8%
Biliary tract infections	25	4.6%
Suspected systemic infections without identified focus ^c^	25	4.6%
Sepsis ^d^	22	4.1%
Bone and joint infections	17	3.2%
Ear, nose and throat infections	15	2.8%
Central nervous system infections	11	2.0%
Neutropenic fever	10	1.9%
Other	5	0.9%
No infectious disease diagnosis	4	0.7%
Total number of patients with SIRS ^e^	256	47.5%
Charlson comorbidity index [15] (mean +/-SD)	2.4 (+/- 2.3)	
McCabe index [16] - not fatal	443	82.2%
McCabe index - fatal < 5 years	64	11.9%
McCabe index - fatal < 6 months	32	5.9%
Died in hospital	31	5.8%

^a ^If patients were on more than one ward, each ward was counted separately.

^b ^Includes gastroenteritis, diverticulitis, peritonitis, enterocolitis, appendicitis, abdominal abscess, acute abdomen.

^c ^These patients did not meet the criteria of the sepsis definition.

^d ^Sepsis: defined as clinical evidence of infection, plus SIRS^e^.

^e ^Systemic inflammatory response syndrome (SIRS): includes two or more of the following: temperature > 38°C or < 36°C, heart rate > 90 beats per minute, leukocytes > 12,000/μL or < 4,000/μL (band forms were not routinely determined and were therefore not considered), respiratory rate > 20 per minute.

### Bacteriological investigations

Within 48 hours of admission a total of 716 bacteriological samples were taken from 438 patients (81.3%). The most commonly performed investigations were blood cultures (333 patients, 61.8% of all patients), urine cultures (181, 33.6%) and cultures of sputum or broncho-alveolar lavage (86, 16.0%). Two-hundred and fourteen pathogenic bacterial isolates were identified in 183 patients. The most common were *Escherichia coli *(64 patients, 12%), *Staphylococcus aureus *(27 patients, 5%), *Streptococcus pneumoniae *(23 patients, 4%), *Klebsiella pneumoniae *(12 patients, 2.2%), *Haemophilus influenza*e (9 patients, 1.7%) and *Pseudomonas aeruginosa *(9 patients, 1.7%).

### Antibiotic therapies

The most commonly used antibiotic for both empirical and adjusted therapy was amoxicillin/clavulanate (Table 2). Broad-spectrum antibiotics (cefepime, imipenem, meropenem, piperacillin/tazobactam) or vancomycin were initially administered to 95 patients (17.6%). Empirical antibiotic therapies lasted a mean of 7.5 days after admission. 421 (83.7%) patients received at least one antibiotic intravenously. Empirical intravenous antibiotics were switched to oral administration a median of 4 days after admission (range: 0 – 43; Interquartile range: 3). The empirical therapy was adjusted in 168 patients (31.2% of all included patients) (Table 2). Of these, 65 patients (38.7%) had their antibiotics switched because of bacteriological results.

**Table 2:** Most frequently used empirical and adjusted antibiotic therapies

Type of therapy	Number of patients	%
**Empirical antibiotic therapies**	**539**	**100%**
Amoxicillin/clavulanate	223	41.4%
Ciprofloxacin	88	16.3%
Piperacillin/tazobactam	55	10.2%
Amoxicillin/clavulanate + clarithromycin	33	6.1%
Ceftriaxone	23	4.3%
Cefepime + amikacin	18	3.3%
Amoxicillin/clavulanate + amikacin	12	2.2%
Ciprofloxacin + metronidazole	9	1.7%
Trimethoprim/sulfamethoxazole	6	1.1%
Norfloxacin	6	1.1%
Cefepime	5	0.9%
Ceftriaxone + clarithromycin	5	0.9%
Other antibiotics or antibiotic combinations	56	10.4%
		
**Adjusted antibiotic therapies**	**168**	**100%**
Amoxicillin/clavulanate	29	17.3%
Piperacillin/tazobactam	22	13.1%
Ciprofloxacin	15	8.9%
Penicillin	12	7.1%
Amoxicillin/clavulanate + ciprofloxacin	8	4.8%
Amoxicillin/clavulanate + clarithromycin	6	3.6%
Meropenem	5	3.0%
Cefuroxime	5	3.0%
Other antibiotics or antibiotic combinations ^a^	66	39.3%

^a ^e.g.: ceftriaxone (4 patients), flucloxacillin (3), amoxicillin (3), levofloxacin (3), ciprofloxacin + metronidazole (3), ciprofloxacin + rifampicin (2), cefepime (2), cefepime + tobramycin (2), clindamycin (2), trimethoprim/sulfamethoxazole (2), imipenem/cilastatin (2), vancomycin (2), ceftriaxone + rifampicin (2), cefuroxime + clarithromycin (2).

Empirical antibiotic therapy was inadequate in 121 patients (22.4%) and the adjusted antibiotic therapy was inadequate in 46 of 168 patients (27.4%). In both empirical and adjusted antibiotic therapy the most common reason for inadequacy was the use of antibiotics with a too broad spectrum (Table 3). The second most common reason for inadequate adjusted therapy was the use of antibiotics ineffective against isolated pathogenic bacteria or against bacteria to be expected according to the postulated infections, or the continuation of antibiotic therapy even after negative cultures ruled out an infection (e.g. in the case of assumed urinary tract infections).

**Table 3:** Reasons for inadequacy of empirical and adjusted antibiotic therapies

Reason for inadequacy ^a^	Number of patients	%
**Empirical antibiotic therapies**	**539**	
Inadequate empirical therapy	121	100%
Spectrum too broad	29	24.0%
Inadequate duration	28	23.1%
Insufficient dosage	27	22.3%
Spectrum too narrow	22	18.2%
Wrong spectrum/inadequate use ^b^	21	17.4%
Excessive dosage	3	2.5%
**Adjusted antibiotic therapies**	**168**	
Inadequate adjusted therapy	46	100%
Spectrum too broad	24	52.2%
Wrong spectrum/inadequate use ^c^	12	26.1%
Insufficient dosage	7	15.2%
Inadequate duration	2	4.4%
Excessive dosage	1	2.2%
Spectrum too narrow	1	2.2%

^a ^More than one reason may apply for each patient.

^b ^If antibiotic given covered completely different spectrum than expected bacteria would require or no antibiotic therapy was warranted.

^c ^If bacteriological results had shown that identified bacteria were resistant to antibiotics used, or antibiotic covered completely different spectrum than expected bacteria would require or no antibiotic therapy was warranted.

### Risk factors for inadequate antibiotic therapy

The univariate analysis of characteristics of patients receiving adequate or inadequate therapy is presented in Tables 4 and 5. In the multivariate analysis, inadequate empirical antibiotic treatment was associated with female sex (odds ratio for adequate treatment: 0.47, 95% confidence interval 0.31–0.72), no infectious disease diagnosis (OR 0.08, 95% CI 0.01–0.78), the use of antibiotic therapies containing vancomycin or broad-spectrum antibiotics (cefepime, imipenem, meropenem, piperacillin/tazobactam) (OR 0.42, 95% CI 0.26–0.70) and the presence of renal failure (OR 0.58, 95% CI 0.36–0.92). Adequate adjusted antibiotic treatment was associated with antibiotic switch due to bacteriological results (OR 5.13, 95% CI 2.13–12.37).

**Table 4:** Characteristics of patients receiving adequate or inadequate empirical antibiotic treatment (univariate analysis).

Characteristic	Patients (n) receiving adequate empirical antibiotic treatment	Patients (n) receiving inadequate empirical antibiotic treatment	p-value	OR (95% CI) for adequate therapy
Number of patients	418 (77.6%)	121 (22.4%)		
Women	170 (70.2%)	72 (29.8%)	**< 0.001**	**0.47 (0.31–0.70)**
**Age**				
Median age and range (years)	67 [18–100]	72 [17–97]	0.038*	
< 40 years	74 (77.1%)	22 (22.9%)	0.904	0.97 (0.57–1.64)
41 – 60 yr.	91 (87.5%)	13 (12.5%)	**0.007**	**2.31 (1.12–4.30)**
> 60 years	253 (74.6%)	86 (25.4%)	**0.034**	**0.62 (0.40–0.97)**
**Ward**:				
Medicine/Geriatrics	281 (78.3%)	78 (21.7%)	0.399	1.19 (0.80–1.76)
Surgery	135 (78.9%)	36 (21.1%)	0.733	1.09 (0.70–1.65)
Medical and surgical intensive care	25 (71.4%)	10 (28.6%)	0.408	0.73 (0.34–1.55)
Neurology	1 (25.0%)	3 (75.0%)	**0.040†**	**0.10 (0.01–0.94)**
**Died in hospital**	25 (80.6%)	6 (19.4%)	0.671	1.22 (0.49–3.04)
**Charlson index**:				
Charlson index total 0	110 (81.5%)	25 (18.5%)	0.206	1.37 (0.84–2.24)
Charlson index total 1 – 5	264 (75.9%)	84 (24.1%)	0.205	0.76 (0.49–1.17)
Charlson index total > 5	44 (78.6%)	12 (21.4%)	0.845	1.07 (0.55–2.10)
**McCabe index:**				
Not fatal	343 (77.4%)	100 (22.6%)	0.882	0.96 (0.56–1.64)
Fatal (< 5 years)	52 (81.3%)	12 (18.8%)	0.450	1.29 (0.67–2.51)
Fatal (< 6 months)	23 (71.9%)	9 (28.1%)	0.428	0.73 (0.33–1.61)
**Number of microbiological exams performed:**				
None	78 (77.2%)	23 (22.8%)	0.931	0.98 (0.58–1.64)
One or more	340 (77.6%)	98 (22.4%)	0.931	1.02 (0.61–1.71)
**Infectious diseases consultants involved:**				
Within 24 hours	23 (62.2%)	14 (37.8%)	**0.020**	**0.45 (0.22–0.89)**
Were involved	58 (69.0%)	26 (31.0%)	**0.042**	**0.59 (0.35–0.99)**
**Therapy adjusted**	128 (76.2%)	40 (23.8%)	0.611	0.89 (0.58–1.38)
**Hepatic failure reported**	1 (50.0%)	1 (50.0%)	0.399†	0.29 (0.02–4.64)
**Renal failure reported**	100 (69.0%)	45 (31.0%)	**0.004**	**0.53 (0.35–0.82)**
**Received broad-spectrum antibiotics or vancomycin**	62 (65.3%)	33 (34.7%)	**0.002**	**0.46 (0.29–0.75)**
**Allergy to antibiotics reported**	29 (70.1%)	12 (29.9%)	0.276	0.68 (0.33–1.37)
**Diagnoses**				
Biliary tract infections	19 (76.0%)	6 (24%)	0.849	0.91 (0.36–2.34)
Bone and joint infections	12 (70.6%)	5 (29.4%)	0.553†	0.69 (0.24–1.99)
Central nervous system infections	9 (81.8%)	2 (18.1%)	1.000†	1.31 (0.28–6.14)
Ear, nose and throat infections	11 (73.3%)	4 (26.7%)	0.753†	0.79 (0.25–2.53)
Gastrointestinal infections	59 (73.8%)	21 (26.3%)	0.377	0.78 (0.45–1.35)
Neutropenic fever	10 (100%)	0 (0.00%)	0.127†	N/A
No infectious disease	1 (25.0%)	3 (75.0%)	**0.037†**	**0.09 (0.01–0.92)**
Respiratory tract infections	139 (82.2%)	30 (17.8%)	0.077	1.51 (0.95–2.39)
Sepsis	19 (86.4%)	3 (13.6%)	0.436†	1.87 (0.55–6.44)
Skin and soft tissue infections	48 (81.4%)	11 (18.6%)	0.458	1.30 (0.65–2.58)
Suspected systemic infections without identified focus	19 (76.0%)	6 (24.0%)	0.849	0.91 (0.36–2.34)
Urinary tract infections, prostatitis, epididimytis	83 (73.5%)	30 (26.6%)	0.240	0.75 (0.47–1.21)
Other	11 (68.8%)	5 (31.3%)	0.371†	1.30 (0.21–1.84)
**SIRS present**	199 (77.7%)	57 (22.3%)	0.923	1.02 (0.68–1.53)

Note: The adequacy of treatment was compared against the individual variables with the Chi-squared test with odds ratio production

* Wilcoxon-Mann-Whitney test

† Fisher's Exact test

**Table 5:** Characteristics of patients receiving adequate or inadequate adjusted antibiotic treatment (univariate analysis)

Characteristic	Patients (n) receiving adequate adjusted antibiotic treatment	Patients (n) receiving inadequate adjusted antibiotic treatment	p-value	OR (95% CI) for adequate therapy
Number of patients	121 (72.5%)	46 (27.5%)		
Women	54 (70.1%)	23 (29.9%)	0.534	0.81 (0.41–1.59)
**Age**				
Median age and range (years)	65 [21–97]	70 [19–95]	0.115*	
< 40	22 (78.6%)	6 (21.4%)	0.427	1.48 (0.56–3.93)
41 – 60	29 (80.6%)	7 (19.4%)	0.219	1.76 (0.71–4.35)
> 60	70 (68.0%)	33 (32.0%)	0.099	0.54 (0.26–1.13)
**Ward**:				
Medicine/Geriatrics	85 (72.0%)	33 (28.0%)	0.472	0.77 (0.38–1.56)
Surgery	36 (73.5%)	13 (26.5%)	0.955	0.98 (0.47–2.06)
Medical and surgical intensive care	13 (86.7%)	2 (13.3%)	0.360†	2.45 (0.53–11.28)
Neurology	0 (0.0%)	0 (0.0%)	N/A	N/A
**Died in hospital**	10 (90.9%)	1 (9.1%)	0.293†	4.05 (0.50–32.60)
**Reason for switch**:				
Switch due to bacterial lab results	58 (89.2%)	7 (10.7%)	**< 0.001**	**5.13 (2.13–12.37)**
Switch due to other reason	43 (64.2%)	24 (35.8%)	0.050	0.51 (0.25–1.01)
**Charlson Index**:				
Charlson index total 0	26 (76.5%)	8 (23.5%)	0.557	1.30 (0.54–3.13)
Charlson index total 1 – 5	84 (71.8%)	33 (28.2%)	0.770	0.89 (0.42–1.89)
Charlson index total > 5	11 (68.8%)	5 (31.2%)	0.771†	0.82 (0.27–2.50)
**McCabe index**				
Not fatal	97 (74.0%)	34 (26.0%)	0.380	1.43 (0.64–3.16)
Fatal (< 5 years)	18 (75.0%)	6 (25.0%)	0.763	1.17 (0.43–3.15)
Fatal (< 6 months)	6 (50.0%)	6 (50.0%)	0.055†	0.35 (0.11–1.14)
**Number of microbiological exams performed:**				
None	13 (76.5%)	4 (23.5%)	0.783†	1.26 (0.39–4.10)
One or more	108 (72%)	42 (28%)	0.783†	0.79 (0.24–2.57)
**Infectious diseases consultants involved:**				
Within 24 hours	22 (88.0%)	3 (12.0%)	0.059	3.19 (0.91–11.21)
Were involved	52 (82.5%)	11 (17.5%)	**0.023**	**2.40 (1.11–5.16)**
**Hepatic failure reported**	1 (100.0%)	0 (0.0%)	1.000†	N/A
**Renal failure reported**	34 (70.8%)	14 (29.2%)	0.766	0.89 (0.43–1.88)
**Received broad-spectrum antibiotics or vancomycin**	37 (84.1%)	7 (15.9%)	0.520	1.28 (0.60–2.72)
**Allergy to antibiotics reported**	9 (64.3%)	5 (35.7%)	0.534†	0.66 (0.21–2.08)
**Diagnoses**				
Biliary tract infections	5 (83.3%)	1 (16.7%)	1.000†	1.94 (0.22–17.06)
Bone and joint infections	10 (100.0%)	0 (0.0%)	0.063†	N/A
CNS infections	3 (100.0%)	0 (0.0%)	0.562†	N/A
Ear, nose and throat infections	0 (0.00%)	1 (100%)	0.275†	N/A
Gastrointestinal infections	17 (63.0%)	10 (37.0%)	0.228	0.59 (0.25–1.40)
Urinary tract infections, postatitis, epididimytis	21 (67.7%)	10 (32.3%)	0.52	0.76 (0.33–1.76)
Neutropenic fever	6 (85.7%)	1 (14.3%)	0.675†	2.35 (0.28–20.05)
Respiratory tract infections	34 (60.7%)	22 (39.3%)	**0.016**	**0.43 (0.21–0.86)**
Sepsis	12 (92.3%)	1 (7.7%)	0.116†	4.95 (0.63–39.24)
Skin and soft tissue infections	10 (71.4%)	4 (28.6%)	1.000†	0.95 (0.28–3.18)
Suspected systemic infections without identified focus	5 (71.4%)	2 (28.6%)	1.000†	0.95 (0.18–5.07)
Other	8 (88.9%)	1 (11.1%)	0.447†	3.19 (0.39–26.21)

* Wilcoxon-Mann-Whitney test

† Fisher's Exact test

## Discussion

The main findings of this study were that I) 19% of patients admitted through the emergency department received antibiotics empirically. II) Empirical antibiotic therapy was inadequate in 22% and adjusted antibiotic therapy in 27% of cases. III) The main reason for inadequacy was the use of antibiotics with an unnecessarily broad spectrum. Finally, IV) we identified risk factors for inadequate empirical antibiotic treatment, such as female sex or renal failure.

### I) Antibiotic utilization rate

We found that 19.4% of medical and surgical patients admitted through the emergency department to a Swiss university hospital are started on therapeutic antibiotics within 24 hours of admission. This antibiotic utilization rate is similar to rates reported for medical admissions to an Acute Medicines Assessment Unit in Aberdeen, Scotland (17%) [8], and to the prevalence of in-patients treated with antibiotics in a Norwegian university hospital (16.6%) [17] and in 8 Swiss non-university hospitals (25%) [9]. Higher antibiotic utilization rates (45.5%) have been previously reported for example from Italian hospitals [18]. However, 84% of patients started on antibiotics in the present study received them in intravenous form, compared to 60% of patients in the Aberdeen study [8].

### II) Inadequacy of antibiotic therapies

The empirical antibiotic therapy was inadequate in 22.4% of our patients. The rate of inadequate treatments among adjusted antibiotic therapies was even higher: 27.4%. These inadequacy rates compare favourably with published data indicating that as many as 41% to 91% of all antibiotic prescriptions in hospitals are inappropriate [11]. In the Aberdeen study for example, empirical therapy was in accordance with the hospital's antibiotic policy in only 52% of patients [8]. In a recent study in a 650-bed hospital in Cleveland, Ohio, 30% of days of antibiotic therapy were deemed unnecessary [7]. Finally, the study examining the rate of inappropriate antibiotic use at 8 medium-sized Swiss hospitals found that 47% of patients not seen by infectious disease consultants had inappropriate antibiotic treatment [9].

Thus, inadequate use of antibiotics in our patients does not appear to be more frequent than at other institutions, despite the fact that no restrictive measures regarding antibiotic use are in place at our hospital. The relatively low rate of inadequate antibiotic therapies in this study might be explained by several factors. On one hand, patients on antibiotic prophylaxis were excluded from the present study, and inadequacy rates for antibiotic prophylaxis are frequently higher than for therapy [9]. On the other hand, differences in the populations studied and in hospital's characteristics may play a role. In this sense, three characteristics of our hospital may be relevant: first, an infectious diseases consultation service which has access to all wards of the hospital and which provides consultations with an "academic detailing" approach (each consultation is discussed in a direct conversation with the intern or resident in charge, providing a targeted one-on-one education) ; second, a close collaboration between infectious diseases department and microbiology laboratory (e.g. one microbiologist participates in the daily meeting of the infectious diseases department, where all patients seen by the infectious diseases consultants are discussed and all positive blood cultures are reviewed); and third, the clinical liaison between laboratory and ward through the infectious diseases consultants, who follow-up all positive blood cultures contacting the physicians in charge on the ward and providing oral clinical advice and, if requested, written consultation. Indeed, each one of these interventions has been shown to improve the use of antibiotics [13,19,20].

### III) Reasons for inadequate adjusted therapies

Despite the interventions mentioned above, the rate of inadequate adjusted therapies in our study was even higher than the rate of inadequate empirical therapies. The main reason for inadequacy of adjusted therapies was the unnecessary use of broad-spectrum antibiotics. This happened frequently because persistent fever after only 24 to 48 hours of empirical antibiotic therapy was considered to be caused by resistant bacteria, and empirical antibiotics were unnecessarily switched to broad-spectrum antibiotics. The second most common reason for inadequacy was the use of antibiotics that were ineffective against isolated pathogenic bacteria or bacteria to be expected according to the identified focus of infection, or the continuation of antibiotic therapy even after an infection was ruled out by negative culture results (e.g. in the case of postulated urinary tract infections). The failure to adapt therapy to culture results suggests that microbiological investigations had an insufficient impact on the management of patients. A similar problem was observed in the Aberdeen study, where in 55% of patients with clinically significant culture results and an inappropriate empirical regimen, the medication was not changed to a more appropriate antibiotic [8]. Furthermore, researchers at the University of Iowa Hospitals and Clinics have previously shown that the reporting of antibiotic susceptibility testing data of positive blood cultures had only a limited impact on antimicrobial management of patients with bloodstream infections [21]. These observations confirm that the reassessment of empirical therapy after 2–4 days, when most culture results are available and clinical evolution is assessable, is crucial. Counselling of prescribers at this stage appears to be essential and is more likely to be efficacious than at an earlier stage [22,23].

### IV) Risk factors for inadequate therapy

Most factors identified in the present study by multivariate analysis as being associated with adequate or inadequate antibiotic therapy are not surprising. Antibiotic therapy was more frequently inadequate in patients with renal failure (mainly because of difficulties in establishing the correct dose), and in patients where no infectious disease was identified or postulated (and where antibiotics were usually started only because of fever). In contrast, a change of antibiotic therapy according to results of microbiological investigations was associated with adequate therapy. The association between use of broad-spectrum antibiotics or vancomycin and inadequate empirical therapy confirms that the use of these medications is often unjustified. It is more difficult to explain why women were less likely to receive adequate empirical antibiotic treatment than men. Gender differences in the prescription of treatment or prophylaxis have previously been reported, e.g. for cardiovascular diseases and HIV-infection [24-27]. To our knowledge however, only one previous study reported that rates of antibiotic use were influenced by gender (females in one New Zealand town received antibiotics more often than males) [28]. This study found also a strong relationship between socioeconomic status and antibiotic dispensing. In our study, women were slightly older than men (mean age ± standard deviation: 65.5 ± 20.4 years, versus 62.3 ± 19.6), and age over 60 years was associated with inadequate empirical antibiotic treatment in univariate analysis. We cannot speculate on the role of socioeconomic status, since we did not collect information on this factor.

The impact of infectious diseases consultations was considered by recording whether infectious diseases consultants were involved within 24 hours of admission or whether they were involved at all. In the univariate analysis involvement of infectious diseases consultants was significantly associated with inadequate empirical therapy (Tab. 4). This can be explained by the fact that consultation is usually required for patients with more severe or difficult to manage infections or with unclear clinical presentation, who are usually started on empirical antibiotic therapy before the infectious diseases consultation is obtained, in order to avoid delays. Thus, adjusted therapies may show more reliably what the effect of infectious diseases consultations was. Indeed, involvement of infectious diseases consultants was associated with adequate adjusted therapy (Tab. 5) in univariate analysis.

The present study has several limitations. In particular, patients started on antibiotics more than 24 hours after admission or who received only antibiotic prophylaxis, patients admitted directly to the wards, and patients who received therapeutic oral antibiotics as out-patients were not included in the study. In addition, only surgical, medical, geriatric, and neurological patients were evaluated. Also, we did not investigate the relationship between adequacy or inadequacy of treatment and clinical outcomes other than in-hospital death. However, this study has also several strengths. Clinical data were raised and evaluated consistently and prospectively in a large defined patient population, and patients were followed until discharge. Furthermore, a large part of in-patients at our hospital, as at other hospitals, are admitted through the emergency department. Hence, our results are clinically relevant for this setting.

## Conclusion

In conclusion, despite the absence of a restrictive antibiotic policy, the rate of inadequate antibiotic therapies at our hospital was similar to rates reported from other institutions. This suggests that restrictions in the antibiotic use are not an absolutely necessary component of an antibiotic stewardship approach based mainly on multidisciplinary collaboration, education and close clinical liaison with the bacteriology laboratory. Nevertheless, the present study demonstrated a significant overuse of antibiotics, especially broad-spectrum antibiotics and intravenous antibiotics, and an insufficient consideration of microbiological results. Surprisingly, adjusted antibiotic therapies were more frequently inappropriate than empirical therapies. Thus, interventions aiming at improving antibiotic prescribing should focus on both initial empirical therapy and streamlining and adjustment of therapy. Furthermore, the impact of microbiological results on the clinical management of patients should be improved.

## Competing interests

The author(s) declare that they have no competing interests.

## Authors' contributions

JM followed up all patients admitted through the Emergency Department, identified all patients qualifying for the study, prospectively collected the information on each patient, reviewed each patients' chart and drafted the manuscript. JM and SB reviewed together each case report form with the corresponding chart to assess the adequacy of the antibiotic therapy. SB conceived of the study and helped to draft the manuscript. MS and PS performed the statistical analysis. All authors participated in the design of the study, helped to interpret the data and to draft the manuscript. All authors read and approved the final manuscript.

## Financial Support

Bristol-Myers Squibb GmbH, Merck Sharp & Dohme-Chibret AG, GlaxoSmithKline AG, AstraZeneca AG, Wyeth Pharmaceuticals AG, Bayer (Schweiz) AG, Pfizer AG. S.B. was supported by a grant from the Department of Internal Medicine (VFWAWF), University Hospital Basel, Basel, Switzerland.

This study was presented in part at the 15^th ^European Congress of Clinical Microbiology and Infectious Diseases, Copenhagen, Denmark, 2–5 April 2005, Abstract O58.

## Pre-publication history

The pre-publication history for this paper can be accessed here:


